# Anti-Inflammatory, Anticholinesterase, and Antioxidant Potential of Scopoletin Isolated from *Canarium patentinervium* Miq. (Burseraceae Kunth)

**DOI:** 10.1155/2013/734824

**Published:** 2013-06-25

**Authors:** R. Mogana, K. Teng-Jin, C. Wiart

**Affiliations:** Center for Natural and Medicinal Products Research, School of Pharmacy, Faculty of Science, University of Nottingham (Malaysia Campus), Jalan Broga, 43500 Semenyih, Selangor Darul Ehsan, Malaysia

## Abstract

Bioassay guided fractionation of an ethanol extract of leaves of *Canarium patentinervium* Miq. (Burseraceae Kunth.) led to the isolation of scopoletin. The structure of this coumarin was elucidated based on spectroscopic methods including nuclear magnetic resonance (NMR-1D and 2D) and mass spectrometry. Scopoletin inhibited the enzymatic activity of 5-lipoxygenase and acetyl cholinesterase with an IC_50_ equal to 1.76 ± 0.01 **μ**M and 0.27 ± 0.02 mM, respectively, and confronted oxidation in the ABTS, DPPH, FRAP, and **β**-carotene bleaching assay with EC_50_ values equal to 5.62 ± 0.03 **μ**M, 0.19 ± 0.01 mM, 0.25 ± 0.03 mM and 0.65 ± 0.07 mM, respectively. Given the aforementioned evidence, it is tempting to speculate that scopoletin represents an exciting scaffold from which to develop leads for treatment of neurodegenerative diseases.

## 1. Introduction

The pathophysiology of Alzheimer's disease, Parkinson's disease, and amyotrophic lateral sclerosis involves death of neurons upon exposure to excessive amounts of reactive oxygen species (ROS) [1]. Indeed, when ROS overwhelms enzymatic and nonenzymatic antioxidant mechanisms, DNA, lipid, and proteins are denatured compelling thus neuroapoptosis [2]. This offset of antioxidant and oxidant balance in the body is called oxidative stress. Oxidative stress results in the damage of biopolymers including nucleic acids, proteins, polyunsaturated fatty acids, and carbohydrates. Oxidative stress causes serious cell damage leading to a variety of human diseases like Alzheimer's disease, Parkinson's disease, atherosclerosis, cancer, liver disease, diabetes, AIDS, arthritis, immunological incompetence, neurodegenerative disorders, inflammation, and so forth [3, 4].

Inflammation in injured cells are both initiated and maintained by the overproduction of prostaglandins and leukotrienes, which are produced by separate enzymatic pathways, namely, the cyclooxygenase (COX) and lipoxygenase (LOX) pathways, respectively. 5-lipoxygenase, catalysing the oxidation of arachidonic acid, produces 5(S)-hydroxyperoxyeicosatetraenoic acid (5-HETE) which undergoes dehydration, resulting in the formation of leukotriene A_4_ (LTA_4_). Enzymatic hydrolysis of LTA_4_, as well as conjugation with other substances, leads to the formation of inflammatory mediators [5]. Leukotrienes have been identified as mediators of a number of inflammatory and allergic reactions including rheumatoid arthritis, inflammatory bowel disease, atopic dermatitis, psoriasis, chronic urticaria, asthma [6, 7], and allergic rhinitis [8].

Furthermore, oxidative and inflammatory processes are among the pathological features associated with the central nervous system in Alzheimer's disease (AD) [9]. The brain of patients suffering from AD is said to be under oxidative stress as a result of perturbed ionic calcium balances within their neurons and mitochondria [10, 11]. Moreover, there is evidence that acetyl cholinesterase (AChE) inhibitors have an anti-inflammatory role through action against free radicals and amyloid toxicity, as well as through decreasing release of cytokines from activated microglia in the brain and blood [11]. There is an established link between the cholinergic system and inflammation as acetylcholine, the principle neurotransmitter, is reported to attenuate the release of cytokines in the parasympathetic anti-inflammatory pathway by which the brain modulates systemic inflammatory responses to endotoxin [12].

*Canarium patentinervium* Miq. is a rare plant from the family of Burseraceae and genus *Canarium* found in Asia Pacific region previously recorded for its usage in wound healing by the indigenous people of Malaysia [13, 14]. Wound healing involves manifold inflammatory processes of which notably the massive release of leukotrienes from arachidonic acid via the 5-lipoxygenase pathway (5-LOX) and the generation of nitric oxide (NO) from inducible nitric oxide synthase (iNOS) [15, 16]. Of note, nitric oxide (NO) is a free radical, and the generation of cytokines involves a reactive oxygen species (ROS) outburst [17]. Therefore, agents able to block the enzymatic activity of 5-LOX and to scavenge free radical are of immense interest against inflammatory conditions which englobe not only epidermal insults but also neurodegeneration and obesity [18, 19]. Cells produce superoxide anion (O_2_^−^), peroxide anion (HO_2_^−^), and hydroxyl ion (HO^−^) as part of the physiological aerobic metabolism which are quickly scavenged by cytoplasmic antioxidant defense system [17]. However, in the event of ageing or pathologies, the antioxidant defense system is overwhelmed, and cells suffer massive oxidative stress leading eventually to carcinogenesis or apoptosis [2]. In fact, oxidative stress is the main causative factor for cholinergic and dopaminergic neurons apoptosis hence AD and Parkinson's disease (PD) [2, 10]. In addition, there is a growing body of evidence that point to the fact that 5-LOX inhibitors are of immense therapeutic values [6, 7, 20–22]. 

In continuation of our earlier studies on the pharmacological properties of* Canarium patentinervium* Miq. [13, 23, 24], this study investigates the inhibition of 5-lipoxygenase, acetylcholinesterase, and antioxidant capacity of an isolated coumarin, scopoletin. To the best of our knowledge, this is the first comprehensive study on scopoletin isolated from *Canarium patentinervium* Miq. investigating the antioxidant capacity, anti-inflammatory, and anti-acetylcholinesterase activities.

## 2. Materials and Methods

### 2.1. Plant Material

The leaves and barks of* Canarium patentinervium* Miq. were collected from one individual tree from Bukit Putih, Selangor, Malaysia (3°5′24′′N  101°46′0′′E). The plant was identified by Mr. Kamaruddin (Forest Research Institute of Malaysia). A herbarium sample (PID 251210-12) has been deposited in the Forest Research Institute of Malaysia. The leaves were air dried and grinded into small particles using an industrial grinder.

### 2.2. Chemical and Reagents

1,1-Diphenyl-2-picryhydrazyl (DPPH), Trolox (6-hydroxy-2,5,7,8-tetramethylchromon-2-carboxylic acid), 2,2′-Azino-bis(3-ethyl-benzthiazoline-6-sulfonic acid), 2,4,6-tripyridyl-s-triazine (TPTZ), quercetin, *β*-carotene, 5,5′-Dithio-bis(2-nitrobenzoic) acid (DTNB), galanthamine, nordihydroguairetic acid (NDGA), and electric eel acetylcholinesterase (Ache) (Type-VI-S, EC 3.1.1.7) were purchased from Sigma Aldrich. Sodium chloride, ascorbic acid, ferric chloride, and glacial acetic acid were purchased from Systerm. Hexane and chloroform were purchased from Friendemann Schmidt Chemicals. Methanol and ethanol 95%, potassium persulfate powder, ferric chloride, ferrous sulphate, tween 20, and potassium phosphate were purchased from Kollin Chemicals. DMSO was from R&M Marketing, Essex UK. Linoleic acid, acetylthiocholine, and 5-lipoxygenase enzyme (human recombinant) were purchased from Calbiochem.

### 2.3. Extraction and Isolation

Dried and grinded sample of leaves (2.8 kg) were soaked in hexane with the ratio of 1 : 3 parts of sample to solvent for 2 h in a 60°C water bath then filtered and concentrated with a rotary evaporator (Buchi, R-200 Switzerland). This was repeated 3 times. Thereafter the leaves and barks were left to air dry completely for 3 days before repeating the whole process with chloroform and then ethanol, respectively. The yield for the hexane, chloroform, and ethanol extract of leaves were 1.25%, 1.11%, and 6.45%, respectively. The ethanol extract of the leaves (80 g) was then partitioned with petroleum ether, chloroform, and water to yield the respective solvent extracts.

**Figure 1:**
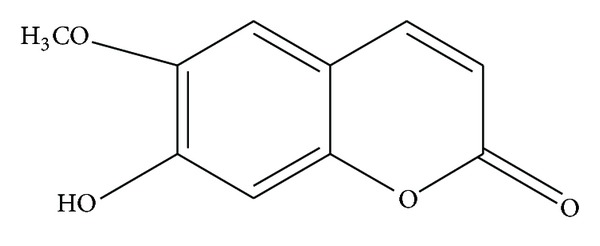
Chemical structure of scopoletin isolated from *Canarium patentinervium* Miq.

The chloroform extract (5 g) was further purified by silica gel chromatography (4 cm × 90 cm, 0.063–0.200 mesh) and eluted with a chloroform/methanol gradient elution (the ratio from 100 : 0 to 8 : 100). Thirteen column fractions were collected and analyzed by TLC (chloroform/methanol). Fractions with similar TLC pattern were combined to total of four fractions. Fraction 2 that was yielded from chloroform/methanol ratio 100 : 4 was rechromatographed on a preparative TLC (2 mm thickness) with solvent system chloroform/methanol (ratio of 1000 : 15) yielding total 7 bands. Band three was collected and rechromatographed on preparative TLC (0.5 mm thickness) with solvent system chloroform/methanol (ratio of 89 : 11) to yield four bands, with band two yielding 7-hydroxy-6 methoxycoumarin (49 mg) also known as scopoletin (Figure 1).

*Scopoletin*. Pale yellow powder; 1H-NMR (400 MHz, CD_3_Cl) *δ*; 3.98 (6-OCH3, s, 3H), 6.30 (H-3, d, *J* = 9.5 Hz, 1H), 6.87 (H-5, s, 1H), 6.95 (H-8, s, 1H), 7.63 (H-4, d, *J* = 9.5 Hz, 1H); 13C-NMR (125 MHz, CD_3_Cl) *δ*; 56.4 (6-OCH3), 103.2 (C-5), 107.4 (C-8), 111.6 (C-3), 113.5 (C-10), 143.3 (C-4), 144.0 (C-6), 149.7 (C-9), 150.2 (C-7), 161.6 (C-2); ESI-MS: *m*/*z* (relative intensity): 192 (M+, 100), 177 (70), 164 (28) 149 (59).

### 2.4. Antioxidant Capacity Tests

Scopoletin was dissolved in dimethyl sulfoxide (DMSO, R&M) prior to assay at a stock concentration of 5 mM, and serial dilution was done accordingly to obtain a good EC_50_ curve. Trolox (6-hydroxy-2,5,7,8-tetramethylchromon-2-carboxylic acid, Sigma-Aldrich), vitamin C (l-ascorbic acid), and quercetin were used as positive control at a stock concentration of 5 mM. Assay was performed using Thermo Scientific Varioskan Flash microtiter plate reader, linked to a computer equipped with (SkanIt Software 2.4.3). EC_50_ values were determined using Prism 5.00 software. At least three independent tests were performed for each sample.

#### 2.4.1. 2,2-Diphenyl-1-picrylhydrazyl (DPPH) Assay

The DPPH assay, as described by Juan-Badaturuge et al. [25], was employed. Aliquots of scopoletin were plated out in triplicate in a 96-well microtiter plate. The 0.1 mM DPPH solution (Aldrich) was added to alternating columns of the test samples and methanol for control of test samples, in the remaining columns. The plate was shaken for 2 min and incubated for 30 min in the dark. The percentage decolourisation was obtained spectrophotometrically at 550 nm. Percentage decolourisation was plotted against the concentration of the sample, and the EC_50_ values were determined using Prism 5.00 software. The DPPH absorbance decreases with an increase in DPPH radical scavenging activity. This activity is given as percent DPPH radical scavenging, which is calculated with the following equation: DPPH radical scavenging activity (%) = [(Abs control − Abs sample)/(Abs control)] × 100, where Abs control is the absorbance of DPPH radical + methanol; Abs sample is the absorbance of DPPH radical + sample extract/standard.

#### 2.4.2. 2,2′-Azino-bis(3-ethyl-benzthiazoline-6-sulfonic acid) (ABTS) Assay

The ABTS assay as described by Miller et al. [26], Rice-Evans [27], and Roberta et al. [28] was employed to determine the radical scavenging activity of the plant extracts. Aliquots of scopoletin were plated out in triplicate in a 96-well microtiter plate at different concentrations. Trolox, vitamin C (l-ascorbic acid), and quercetin were used as positive control and prepared in ethanol, and serial dilutions of this positive control were also prepared. Ethanol was used as the negative control. The stock solution included 7 mM ABTS solution and 2.4 mM potassium persulfate solution. The working solution was then prepared by mixing the two stock solutions in equal quantities. This solution was then stored in the dark for 12–16 hours in order to stabilise it before use. It remains stable for 2–3 days in the dark. The concentrated ABTS^+^ solution was diluted with cold ethanol shortly before conducting the assay, to a final absorbance of 0.70 ± 0.01 at 734 nm at 37°C, in a 3 cm cuvette. The total scavenging capacity of the extracts was quantified through the addition of 100 *μ*L ABTS^+^ to 100 *μ*L of test sample. The solutions were heated to 37°C for 7 min, after which the absorbance was read at 734 nm. The percentage decolourisation was calculated using equation below, and the extent of inhibition of the absorbance of the ABTS^+^ was plotted as a function of the concentration. This activity is given as percent ABTS radical scavenging, which is calculated with the following equation: ABTS radical scavenging capacity (%) = [(Abs control − Abs sample)/(Abs control)] × 100, where Abs control is the absorbance of ABTS radical + ethanol; Abs sample is the absorbance of ABTS radical + sample extract/standard.

#### 2.4.3. Ferric Reducing Ability of Plasma (FRAP) Assay

The antioxidant activity scopoletin was determined using the colorimetric FRAP assay, as described by Benzie and Strain [29] with slight modifications. Aliquots of scopoletin were plated out in triplicate in a 96-well microtiter plate at different concentrations. The working FRAP reagent was prepared just before assay by mixing 300 mM of acetate buffer (pH 3.6), 10 mM of 2,4,6-tripyridyl-s-triazine (TPTZ), and 20 mM of FeCl_3_·6H_2_O in ratio of 10 : 1 : 1. Briefly 180 *μ*L of the FRAP reagent was mixed with 20 *μ*L of the test sample so that the final dilution of the test sample in the reaction mixture was 1/10. After 30 minutes, the absorbance of the coloured product (ferrous triphyridyltriazine complex) was recorded. Fe(II) concentrations in the range of 1 *μ*M–100 *μ*M (FeSO_4_·7H_2_O) was used as standard for calibration curve, and equation of linearity is determined (*y* = *ax* + *b*). From the linearity equation, concentration of sample that produced same absorbance as 1 mM of Fe(II) was determined (*y* of sample filled in equation to obtain *x*). The antioxidant activity was calculated as Ferrous Equivalents, the concentration of samples which produced an absorbance value equal to that of 1 mM FeSO_4_.

#### 2.4.4. *β*-Carotene Bleaching Assay

The *β*-carotene bleaching assay was conducted according to the method described by Habtemariam and Jackson [30] with some modifications. Aliquots of scopoletin were plated out in triplicate in a 96-well microtiter plate at different concentrations. Briefly, 1 mL of a *β*-carotene solution in chloroform (2 mg in 10 mL) was pipetted into a round bottom flask containing 40 *μ*L of linoleic acid and 500 *μ*L of Tween 20. After the removal of chloroform using a rotary vacuum evaporator at 45°C, 100 mL of deionised water were added with vigorous agitation. One hundred eighty microlitres of the emulsion was added to 20 *μ*L of test samples at varying concentrations in 96-well microtiter plate. The absorbance was measured at 470 nm immediately against a blank consisting of the emulsion without *β*-carotene and after 3 h of incubation at 50°C using a spectrophotometer. The antioxidant activity of test agents was evaluated in terms of bleaching of *β*-carotene using the following formula: antioxidant activity AA  (%) = [1 − (*A*_0_ − *A*_*t*_)/(*A*_0_′ − *A*_*t*_′)]  × 100, where *A*_0_ and *A*_0_′ are absorbances measured at zero time of incubation for the test sample and control, respectively; *A*_*t*_ and *A*_*t*_′ are the absorbances measured in the test sample and control, respectively, after incubation for 3 hr.

### 2.5. The 5-Lipoxygenase Inhibition Assay

The 5-lipoxygenase assay was conducted according to the method described by Baylac and Racine [31] with some modifications. Ice-cold buffer (potassium phosphate) at 4°C was mixed with 100 U of the thawed 5-lipoxygenase enzyme. Test sample scopoletin and nordihydroguaiaretic acid (NDGA) which was used as positive control was dissolved in dimethyl sulfoxide (DMSO, R&M) prior to assay at a stock concentration of 5 mM, and serial dilution was done according to the assay to obtain a good EC_50_ curve. Twenty microliters of scopoletin was plated out in triplicate in a 96-well microtiter plate at different concentrations, followed by 160 *μ*L of 0.1 M potassium phosphate buffer (pH 6.3) maintained at 25°C and 20 *μ*L of enzyme solution. Mixture was agitated, and 10 *μ*L of linoleic acid was added and incubated at 10 mins at 25°C. Absorbance was recorded at 234 nm using Thermo Scientific Varioskan Flash microtiter plate reader, linked to a computer equipped with (SkanIt Software 2.4.3). Percentage inhibition of enzyme was determined by comparison of rates of reaction of samples relative to blank sample using the formula (*E* − *S*)/*E* × 100, where *E* is the activity of enzyme without test sample and *S* is the activity of enzyme with test sample. The experiments were done in triplicate. 

### 2.6. Anti-Acetylcholinesterase Assay

Acetylcholinesterase (AChE) inhibitory activity was measured by slightly modifying the spectrophotometric method developed by Ellman et al. [32]. 5,5′-Dithio-bis(2-nitrobenzoic) acid (DTNB, Sigma, St. Louis, MO, USA) was used for the measurement of anti-AChE activity. All the other reagents and conditions were same as described previously [9]. Test sample scopoletin and galanthamine which was used as positive control was dissolved in dimethyl sulfoxide (DMSO, R&M) prior to assay at a stock concentration of 5 mM, and serial dilution was done accordingly to obtain a good EC_50_ curve. In brief, 130 *μ*L of 0.1 mM sodium phosphate buffer (pH 8.0), 20 *μ*L of DTNB, 20 *μ*L of test solution, and 20 *μ*L of AChE solution were added by multichannel automatic pipette (Eppendorf, Germany) in a 96-well microplate and incubated for 15 min at 25°C. The reaction was then initiated with the addition of 10 *μ*L of acetylthiocholine iodide. The hydrolysis of acetylthiocholine iodide was monitored by the formation of the yellow 5-thio-2-nitrobenzoate anion as a result of the reaction of DTNB with thiocholines, catalyzed by enzymes at a wavelength of 412 nm utilizing a 96-well microplate Thermo Scientific Varioskan Flash microtiter plate reader, linked to a computer equipped with (SkanIt Software 2.4.3). Percentage inhibition of AChE was determined by comparison of rates of reaction of samples relative to blank sample (ethanol in phosphate buffer pH = 8) using the formula (*E* − *S*)/*E* × 100, where *E* is the activity of enzyme without test sample and *S* is the activity of enzyme with test sample. The experiments were done in triplicate. Galanthamine was purchased from Sigma (St. Louis, MO, USA) and used as reference.

### 2.7. Statistical Analysis

Concentration-response curves were calculated using the Prism software package 5.00 for Windows, GraphPad Software, San Diego, California, USA, http://www.graphpad.com/ (GraphPad, San Diego, USA), and data were obtained from three independent experiments, each performed in triplicates (*n* = 9) and represented as mean ± SD. Nonlinear best fit was plotted with mean ± SD. One way Anova was performed followed by Tukey's multiple comparison tests. Throughout the analysis, *P* < 0.05 was considered significant. 

## 3. Results and Discussions

### 3.1. Antioxidant Capacity

*In vitro* antioxidant capacity can be determined by hydrogen atom transfer (HAT) method and single electron transfer (SET) method [4]. HAT based methods measure the ability of an antioxidant to scavenge free radical by hydrogen donation to form a stable compound. SET based methods detect the ability of the antioxidant to transfer one electron to reduce compound including metals, carbonyls and radicals [33, 34]. *β*-carotene bleaching assay involves HAT method, FRAP assay involves SET method, while DPPH and ABTS assay involves both method predominantly via SET method [3, 35].

FRAP is the ferric reducing power of antioxidants by the reduction of the ferric ions to the ferrous ions, which form a blue coloured ferrous-tripyridyltriazine complex (ferric TPTZ) which is detected at 593 nm. Deeper blue colour indicates higher antioxidant potential [36]. The compounds absorbance equivalent to 1 mM FeSO_4_ was calculated from equation of linearity of ascorbic acid (*y* = 0.0301*x* + 0.0831, *r* ^2^ = 0.9799), trolox (*y* = 0.1193*x* + 0.2815, *r* ^2^ = 0.9904), quercetin (*y* = 0.3053*x* + 0.4857, *r* ^2^ = 0.9646), and scopoletin (*y* = 0.0203*x* + 0.5259, *r* ^2^ = 0.9837). Total FRAP value was determined from the absorbance value above using the standard Fe(II) calibration curve equation (*y* = 0.0105*x* + 0.0136, *r* ^2^ = 0.9817) (figure not shown). Scopoletin displayed significant (*P* < 0.05) FRAP value (254.99 ± 0.64 *μ*M) compared to ascorbic acid (1970.00 ± 0.23 *μ*M).

In the *β*-carotene/linoleic model, linoleic acid reacts with ROS and O_2_ to form an unstable peroxy radical. *β*-carotene being an antioxidant will react with this radical to form stable epoxide causing the bleaching of yellow solution. Competition reaction occurs with the presence of another antioxidant (sample) to react with the peroxy radical resulting in slower bleaching of solution detected at 470 nm spectrophotometrically [37]. Scopoletin displayed a moderate antioxidant activity with an EC_50_  647.89 ± 0.07 *μ*M.

DPPH assay is based in the ability of antioxidant to reduce stable DPPH radical to form yellow coloured *α*, *α*-diphenyl-*β*-picryl hydrazine thus decolourising the deep purple DPPH methanol solution. Greater discolourisation results in lower absorbance at 550 nm indicating higher antioxidant capacity [25, 36]. Scopoletin exhibited moderate antioxidant activity with an EC_50_ = 191.51 ± 0.01 *μ*M. ABTS assay involves the reduction of the blue-green 2,2-azino-bis(3-thylbenzothiazoline-6-sulfonate) radical cation (ABTS^*∙*+^) by antioxidants to its original colourless ABTS form. Greater discolourisation results in lower absorbance at 734 nm indicating higher antioxidant capacity [26, 27]. Scopoletin displayed significant (*P* < 0.05) EC_50_ (5.62 ± 0.03 *μ*M) as opposed to ascorbic acid (8.74 ± 0.06 *μ*M). The DPPH and ABTS assays have the same mechanism of action, but, in most cases, the results obtained from the ABTS assay are higher than those from DPPH assay. It has been documented that results reported for the ABTS assay do not only take into account the activity of the parent compound, but also the contribution of reaction products and other individual compounds on the activity, which is not the case in the DPPH assay [38, 39].

Isolated compound scopoletin showed significantly lower FRAP value (254.99 ± 0.64 *μ*M) compared to quercetin and ascorbic acid and lower ABTS value (5.62 ± 0.03 *μ*M) compared to ascorbic acid (Table 1). It can be concluded that scopoletin exhibits its antioxidant activity predominantly via the SET method.

### 3.2. Anti-Inflammatory Activity

This assay measures the inhibitory activity against 5-LOX enzyme, which is the key enzyme in the metabolism of arachidonic acid that is responsible for the formation of leukotrienes which play a pivotal role in the pathophysiology of chronic inflammatory and allergic diseases. 5-Lipoxygenase is known to catalyse oxidation of unsaturated fatty acids containing 1-4-diene and the modification of linoleic acid (1-4-diene into 1-3-diene) can be detected at 234 nm.

In the 5-LOX assay, scopoletin displayed potent enzyme inhibition (IC_50_ = 1.76 ± 0.01 *μ*M) which was fiftyfold more than nordihydroguaiaretic acid (IC_50_ = 85.23 ± 0.02 *μ*M) (Table 2). According to the 5-LOX enzyme inhibition activity measurement [39], scopoletin displays good enzyme inhibition activity with IC_50_ of 0.34 ± 0.01 *μ*g/mL. (IC_50_ < 30 *μ*g/mL: good activity; 30 < IC_50_ < 80 *μ*g/mL: moderate activity; IC_50_ > 80 *μ*g/mL: poor activity). A combination of anti-inflammatory and antioxidant assays constitutes a good indication on potential anti-inflammatory activity of a drug [40, 41], as inhibition of the lipoxygenases is due to reaction of the inhibitor with free radicals generated at the active site of the enzyme [42]. 

### 3.3. Anti-AChE Activity

This assay measures the inhibition activity against AChE, which is the key enzyme in the hydrolysis of acetylcholine that is responsible for muscle and organ relaxations. Acetylcholinesterase inhibitors are therefore used medicinally to treat myasthenia gravis to increase neuromuscular transmission and to treat Alzheimer's disease (deficiency in the production of acetylcholine).

In the anti-AChE assay, scopoletin reported a moderate activity (IC_50_ = 270.00 ± 0.02 *μ*M) compared to galanthamine (IC_50_ = 2.57 ± 0.06 *μ*M) as in Table 2. 

## 4. Conclusion

Coumarins (known as 1,2-benzopyrones) consisting of fused benzene and pyrone ring, are an important group of low-molecular weight phenolics and have been widely used for prevention and treatment of various diseases. Hydroxycoumarins have attracted intense interest in recent years because of their diverse pharmacological properties. In particular, natural source of 7-hydroxycoumarins have been studied for their antioxidant and anti-inflammatory actions [43]. Drug discovery from natural sources is an area pertinent to complementary and alternative medicine (CAM) [44]. Scopoletin has been studied to have good pharmacokinetics data with good absorption in the stomach and colon of rats [45]. Reported physical properties of scopoletin which passes the Lipinsky rule (mass < 500, log *P* < 5, donor count < 5, and acceptor count < 10), for possible lead compound in drug discovery [46] and, in agreement with its potent antioxidant power, good anti-inflammatory and moderate anti-acetylcholinesterase activity demonstrated in this study might be of value for the treatment of various diseases emerging from oxidative stress and thus warrants further *in vivo *studies. Given the aforementioned evidence it is tempting to speculate that scopoletin represents an exciting scaffold from which to develop leads for treatment of neurodegenerative diseases.

## Figures and Tables

**Table 1:** Antioxidant capacity of scopoletin isolated from *Canarium patentinervium* Miq.

Extracts	ABTS assay, EC_50_ (*µ*M)	DPPH assay, EC_50_ (*µ*M)	FRAP assay, FRAP value (*µ*M)	*β*-carotene bleaching assay, EC_50_ (*µ*M)
SC	5.62 ± 0.03	191.51 ± 0.01	254.99 ± 0.64	647.89 ± 0.07
AA	8.74 ± 0.06	10.68 ± 0.01	1970.00 ± 0.23	NA
QC	2.91 ± 0.03	9.52 ± 0.02	284.00 ± 0.24	5.42 ± 0.04^a^
TRO	2.72 ± 0.02	19.06 ± 0.04	131.85 ± 0.54	6.59 ± 0.03^a^

AA: ascorbic acid, QC: quercetin, TRO: Trolox, and SC: isolated compound scopoletin. Data was obtained from three independent experiments, each performed in triplicates. Values with the same letter are not significantly different (*P* < 0.05) according to Tukey multiple comparison test.

**Table 2:** Anti-inflammatory and antiacetylcholinesterase values of scopoletin isolated from *Canarium* *patentinervium* Miq.

Compound	Anti-inflammatory assay 5-LOX, IC_50_ (*µ*M)	Antiacetylcholinesterase assay, IC_50_ (*µ*M)
SC	1.76 ± 0.01	270.00 ± 0.02
NDGA	85.23 ± 0.02	NA
Galantamine	NA	2.57 ± 0.06

SC: isolated compound scopoletin, NDGA: nordihydroguaiaretic acid, NA: not applicable.

Data were obtained from three independent experiments, each performed in triplicates. (*n* = 9) and represented as mean ± SD.

IC_50_ < 30 *µ*g/mL: good activity; 30 < IC_50_ < 80 *µ*g/mL: moderate activity; IC_50_ > 80 *µ*g/mL: poor activity.
